# Distribution of Mesenchymal Stem Cells and Effects on Neuronal Survival and Axon Regeneration after Optic Nerve Crush and Cell Therapy

**DOI:** 10.1371/journal.pone.0110722

**Published:** 2014-10-27

**Authors:** Louise Alessandra Mesentier-Louro, Camila Zaverucha-do-Valle, Almir Jordão da Silva-Junior, Gabriel Nascimento-dos-Santos, Fernanda Gubert, Ana Beatriz Padilha de Figueirêdo, Ana Luiza Torres, Bruno D. Paredes, Camila Teixeira, Fernanda Tovar-Moll, Rosalia Mendez-Otero, Marcelo F. Santiago

**Affiliations:** 1 Instituto de Biofísica Carlos Chagas Filho, Universidade Federal do Rio de Janeiro, Rio de Janeiro, Brazil; 2 Instituto Nacional de Ciência e Tecnologia de Biologia Estrutural e Bioimagem, INBEB, Rio de Janeiro, Brazil; 3 National Center of Structural Biology and Bioimaging (CENABIO), Universidade Federal do Rio de Janeiro, Rio de Janeiro, Brazil; 4 D'Or Institute for Research and Education (IDOR), Rio de Janeiro, Brazil; 5 Institute of Biomedical Sciences (ICB), Universidade Federal do Rio de Janeiro, Rio de Janeiro, Brazil; Universidade de Sao Paulo, Brazil

## Abstract

Bone marrow-derived cells have been used in different animal models of neurological diseases. We investigated the therapeutic potential of mesenchymal stem cells (MSC) injected into the vitreous body in a model of optic nerve injury. Adult (3–5 months old) Lister Hooded rats underwent unilateral optic nerve crush followed by injection of MSC or the vehicle into the vitreous body. Before they were injected, MSC were labeled with a fluorescent dye or with superparamagnetic iron oxide nanoparticles, which allowed us to track the cells *in vivo* by magnetic resonance imaging. Sixteen and 28 days after injury, the survival of retinal ganglion cells was evaluated by assessing the number of Tuj1- or Brn3a-positive cells in flat-mounted retinas, and optic nerve regeneration was investigated after anterograde labeling of the optic axons with cholera toxin B conjugated to Alexa 488. Transplanted MSC remained in the vitreous body and were found in the eye for several weeks. Cell therapy significantly increased the number of Tuj1- and Brn3a-positive cells in the retina and the number of axons distal to the crush site at 16 and 28 days after optic nerve crush, although the RGC number decreased over time. MSC therapy was associated with an increase in the FGF-2 expression in the retinal ganglion cells layer, suggesting a beneficial outcome mediated by trophic factors. Interleukin-1β expression was also increased by MSC transplantation. In summary, MSC protected RGC and stimulated axon regeneration after optic nerve crush. The long period when the transplanted cells remained in the eye may account for the effect observed. However, further studies are needed to overcome eventually undesirable consequences of MSC transplantation and to potentiate the beneficial ones in order to sustain the neuroprotective effect overtime.

## Introduction

Diseases that affect the optic nerve, such as glaucoma and diabetic retinopathy, are common causes of blindness worldwide [1]. In addition, traumatic optic neuropathy leads to visual impairment and frequently to irreversible blindness [2]. Visual loss occurs because, in mammals, injury to the optic nerve, e.g., crush or transection, results in the progressive retrograde degeneration of axons and the death of retinal ganglion cells (RGC), mainly by apoptosis [3]–[5].

Strategies developed to enhance survival and regeneration of RGC include the inhibition of myelin-derived proteins and blockage of rho kinase [6]–[9], deletion of PTEN [10] and/or SOCS-3 [11], [12], macrophage activation and delivery of oncomodulin [13]–[18], delivery and stimulation of ciliary neurotrophic factor [8], [19], [20], regulation of KLF family members [21], cell therapy [22]–[24] and a combination of multiple approaches [14], [25]. Despite the remarkable progress in the understanding of the mechanisms and pathways involved in neuronal survival and regeneration, at present there are no clinically and currently applicable therapies to sustain RGC survival and/or to promote long-distance axon regeneration. Injection of trophic factors into the vitreous body prevents neuronal loss, but the effect is transitory [26], and even after peripheral-nerve grafting, which provides a permissive environment for regeneration of central neurons, RGC survival decreases overtime [27].

Cell therapy with bone marrow-derived cells is a potentially useful approach since these cells can be used as a source of trophic factors [28], have immunomodulatory properties [29], and can be transfected to enhance the production of specific factors [30]. The bone marrow is the best-characterized source of adult stem cells [31], which have been widely used in models of neurological diseases [32], such as brain ischemia [33]–[36], spinal cord injury [37], peripheral nerve injury [38], and in the visual system, in models of glaucoma [22] and optic nerve injury [23], [24], [39].

Of importance, homing of bone marrow cells after transplantation might be crucial, since they are attracted to damaged areas of the nervous system [40]. Several studies have analyzed short-term engrafting of mesenchymal stem cells (MSC) after transplantation into the eye, using *in vitro* and *ex vivo* approaches [41]–[44]; but, to our knowledge, there are no reports of long-term *in vivo* tracking of MSC injected into the eye after optic nerve injury.

In this study, we investigated whether MSC can protect RGC from death and increase axonal regeneration in a model of optic nerve crush. In addition, for the first time, we followed transplanted MSC labeled with superparamagnetic iron oxide nanoparticles (SPION) *in vivo* during several weeks, using magnetic resonance imaging (MRI).

## Materials and Methods

### Animals and ethics statement

A total of 61 adult (3-5-month-old) Lister Hooded rats were used in this study. Animals were used in accordance with the ARVO Statement for the Use of Animals in Ophthalmic and Vision Research, and the protocols were approved by the Committee for the Use of Experimental Animals of the Centro de Ciências da Saúde from the Universidade Federal do Rio de Janeiro (reference number: DAHEICB 052). Seven rats were used for extraction of bone marrow cells, and 54 for optic nerve crush. All procedures were performed under anesthesia with ketamine and xylazine or isofluorane, and every effort was made to minimize suffering.

### Bone marrow extraction and cell culture

Adult Lister Hooded rats were deeply anesthetized by inhalation of isofluorane and euthanized by cervical dislocation. Bone marrow was extruded from femurs and tibias and seeded into plastic culture flasks at a density of 1×10^6^ cells/cm^2^ in DMEM-F12 containing 10% fetal bovine serum (FBS), penicillin (100U/mL) and streptomycin (100 µg/mL, all from Invitrogen Inc., Carlsbad, CA, USA) and kept in an incubator at 37°C and 5%/95% CO_2_/air. After 48 h, non-adherent cells were removed by washing with phosphate-buffered saline (PBS) and the medium was completely changed every 2–3 days. Cells were grown until approximately 90% confluent and then passaged (0.25% trypsin 1 mM EDTA, Invitrogen Inc.) and plated at a density of 7×10^3^ cells/cm^2^. For cryopreservation, cells were frozen at the first or second passages in FBS with 10% dimethyl sulfoxide (DMSO; Merck KGaA, Darmstadt, Germany), using a Bio-Cool Controlled Rate Freezer (SP Scientific, Warminster, PA, USA), and then stored in liquid nitrogen. For all the experiments, thawed cells were cultured and used from the third to fifth passages.

### Multipotent mesenchymal stem cell characterization

MSC identity was analyzed by the induction of adipogenesis, osteogenesis and chondrogenesis, and also by immunophenotyping.

MSC differentiation assays were performed as described previously [45]. Briefly, to evaluate the adipogenic differentiation, ∼50% confluent cells were cultivated for 16 days in DMEM F-12 supplemented with 1 µM dexamethasone, 10 µg/mL insulin, 0.5 µM isobutylmethylxanthine and 200 µM indomethacin. Cells were stained with 0.2% Oil Red O for 30 min to reveal the intracellular accumulation of lipid-rich vacuoles. Osteogenic differentiation was performed with medium supplemented with 1 µM dexamethasone, 10 mM b-gly-cerolphosphate, and 0.5 µM ascorbic phosphate. Calcium deposits were revealed by 1% Alizarin Red staining for 30 min in water. All reagents used for both assays were from Sigma (Sigma-Aldrich Co., St Louis, MO, USA). To investigate the chondrogenic differentiation potential, labeled MSCs were trypsinized and resuspended in culture medium at 1.6×10^7^ cells/mL. To form micromass cultures, cells were seeded in 7-µL droplets in the center of 24-well plates and cultured under high-humidity conditions. After 2 h chondrogenic medium (Invitrogen Inc.) was added to the culture plates and the cells were cultured for 2 weeks. The micromass formed was embedded in paraffin, sectioned, and the presence of proteoglycans was evaluated by staining with 1% Alcian Blue (Sigma-Aldrich Co.) in 3% acetic acid (Sigma-Aldrich Co.) solution for 30 min.

MSC immunophenotyping was determined by the presence or absence of characteristic surface markers [31], [46]. For flow cytometry analysis, 5×10^6^ cells were suspended in 500 µL saline and incubated with rat immunoglobulins and 5% FBS for 20 min at room temperature, to block unspecific binding. Aliquots of 1×10^6^ cells were incubated for 20 min in the dark, at 4°C, with the following antibodies: anti-CD34-PE (1∶100, Santa Cruz Biotechnology, Santa Cruz, CA, USA), anti-CD45-FITC, anti-CD11b-FITC (1∶100, both from Invitrogen Inc.), anti-CD90-PE and anti-CD29-FITC (1∶100, both from BD Biosciences, San José, CA, USA). Aliquots of 5×10^5^ cells were incubated without antibodies as a control, or with propidium iodide to evaluate cell viability. Cells were washed twice in PBS and analyzed in a BD FACS Aria II flow cytometer (BD Biosciences). Acquired data were analyzed by FlowJo v.7.6.4 software (FlowJo, USA).

### Preparation of MSC for transplantation

On the day of the transplantation, MSC were detached from the plates using trypsin-EDTA and centrifuged at 300×g for 5 min, with medium containing 10% FBS. Precipitated cells were suspended in PBS containing DNAse (625 ng/mL, dornase alpha-rhDNase, Pulmozyme, Roche), to avoid aggregation of DNA released by disrupted cells, and centrifuged again. Cells were counted in an Automated Cell Counter (Countess Automated Cell Counter, Invitrogen Inc.) and suspended at a concentration of 1×10^5^ cells/µL in saline containing DNAse prior to administration.

### MSC labeling

Cells were labeled before transplantation to allow tracking by confocal microscopy or by MRI. For the short period of evaluation of the transplant, cells were incubated in suspension with CellTrace FarRed DDAO-SE (2.5 µg/mL) for 40 min at 37°C and 5%/95% CO_2_/air. CellTrace strongly binds to primary amines inside and outside the cell, according to the manufacturer. Cells were washed three times in PBS and prepared for administration as described previously. For long-term and *in vivo* tracking of the transplanted cells, they were incubated with superparamagnetic iron oxide (SPION) particles (FeraTrack Contrast Particles, Milteny Biotec, Bergisch Gladbach, Germany), as previously described [45]. Briefly, particles were prepared as described by the manufacturer and incubated with the cells in culture for 4 h, at 37°C and 5%/95% CO_2_/air. The medium was removed, and the cells were washed with PBS, detached with trypsin-EDTA and prepared for administration as described above.

Incorporation of FeraTrack nanoparticles by the MSC was analyzed by immunocytochemistry to identify the dextran-coating of the particles, and/or by Prussian blue reaction to detect iron within the cells, as described previously [45]. For these analyses the labeled cells were plated on glass coverslips coated with 0.1% gelatin, rinsed in PBS and fixed in 4% paraformaldehyde.

For immunocytochemistry, coverslips were rinsed in 0.1% Triton X-100 in PBS (PBST) and incubated in 5% normal goat serum (Sigma-Aldrich Co) for 30 min at room temperature. The cells were incubated in primary antibody (anti-dextran, 1∶500, StemCell Technologies, Vancouver, BC, Canada) overnight at 4°C. Cells were then washed in 0.1% PBST and incubated with the secondary antibody (Alexa Fluor 488 goat anti-mouse IgG, 1∶1000, Invitrogen Inc.) and TO-PRO-3 stain to label nuclei (1∶1000, Invitrogen Inc.) for 2 h at room temperature. Coverslips were rinsed in PBS and mounted with Vectashield (Vector Laboratories, Burlingame, CA). Fluorescent samples were analyzed under a confocal microscope (Zeiss LSM 510 Meta). For the Prussian blue reaction, cells were washed twice with PBS and incubated with Perls' reagent (20% potassium ferrocyanide and 20% hydrochloric acid in water) for 20 min at room temperature. Cultures were then washed once in PBS, dehydrated through a graded ethanol series and mounted with Entellan (Merck KGaA). The samples were observed by light microscopy.

### Optic-nerve crush and intraocular injections

A total of 54 animals were submitted to optic nerve crush, performed as previously described [39]. Briefly, adult Lister-hooded rats were anesthetized by intraperitoneal injection of ketamine (50 mg/Kg) and xylazine (15 mg/Kg). Under a stereoscopic microscope, the left optic nerve was accessed by making an incision in the skin covering the orbital bone. The nerve was exposed and the dura mater surrounding it was cut longitudinally. Nerve crush was performed by compression with tweezers for 15 s, at 1 mm behind the eye, with care to avoid damage to the blood vessels. Immediately after the crush, a 5 µL suspension of 5×10^5^ MSC or vehicle (0.9% saline with DNAse) was injected into the vitreous, in the intersection between the sclera and the cornea at the upper temporal side of the eye, with care to avoid lens injury. The same volume of vitreous was aspirated before injection of cells or vehicle, in order to avoid increasing the pressure inside the eye. Before and after the procedure, the back of the eye was observed through the operating dissecting microscope as previously described [23], [39] to assess the integrity of the retinal blood flow. Animals with damage to the lens or to the retinal blood vessels were excluded from the experiment. After the procedure, the incision in the skin was sutured and topical anesthetic was applied. Animals were kept under supervision and warmed by a lamp until they recovered from the anesthesia.

### *In vivo* magnetic resonance imaging (MRI)

Cells were tracked in vivo by MRI measurements at 2, 14 and 18 weeks after optic nerve crush and cell transplantation. MRI experiments were performed in the National Center of Structural Biology and Bioimaging (CENABIO/UFRJ). The right eye was used as a control and was injected intravitreously with the vehicle of the cells. For MRI, animals were anesthetized by intraperitoneal injection of ketamine (50 mg/Kg) and xylazine (15 mg/Kg) and positioned in the MRI coil. Images were acquired in a 7-T magnetic resonance scanner (7T/210 horizontal Varian scanner, Agilent Technologies, Palo Alto, CA, USA), using fast spin echo (FSE) proton density (PD) sequences (matrix: 192×192, slice thickness: 0.5 mm; 15 continuous slices) in the axial (TR/TE: 1500/11 ms; field of view: 30×30 cm), coronal (TR/TE: 2100/11 ms; field of view: 30×30.5 cm) and sagittal (TR/TE: 1500/11 ms; field of view: 30×30.5 cm) planes. Data were processed with the use of VnmrJ Software (Agilent Technologies).

### RGC survival analysis

Animals were euthanized with an overdose of anesthetics and perfused through the heart with ice-cold saline, followed by 4% paraformaldehyde in 0.1 M phosphate buffer. Eyes were removed and retinas were dissected in PBS. Free-floating retinas were frozen in 0.5% PBST for 15 min at −80°C. Retinas were thawed and washed twice in the same solution for 10 min at room temperature. Primary antibodies anti-Brn3a produced in goat (1∶250, Santa Cruz Biotechnology) and anti-Tuj1 produced in mouse (1∶250, Covance, Berkeley, CA, USA) were diluted in 0.2% PBST and 5% normal donkey serum and incubated overnight at 4°C. Retinas were washed three times in PBS and incubated with the secondary antibodies Cy3 donkey anti-goat IgG (1∶1000, Jackson Immunoresearch Laboratories, West Grove, PA) and Alexa Fluor 488 donkey anti-mouse IgG (1∶500, Invitrogen Inc.) in the same solution of the primaries for 2 h at room temperature. Retinas were washed three times in PBS, flat-mounted, and covered with Vectashield (Vector Laboratories). All steps were performed under gentle shaking, except the freezing procedure.

Retinas were imaged under a Zeiss Axiovert 200 M microscope equipped with epi-fluorescence optics and a Zeiss MRM digital camera, at 1.0 and 3.5 mm from the optic disc, in all quadrants of the retina. For Brn3a, 5 images of 0.135 mm^2^ were taken at each distance, and for Tuj1, 10 images of 0.032 mm^2^ were taken at each distance. Tuj1 images were manually counted by a blinded observer. Brn3a images were automatically counted using the Analyze Particles function of Image J software (NIH); before opting for the automatic cell count, we compared manual and automatic counts and found no statistically significant difference (data not shown). The mean number of cells was divided by the area of the image to estimate the number of RGC per square millimeter of retina. The number of cells in each experimental group was normalized to the control group (contralateral eyes) and expressed as a percentage of the control. To estimate the total number of cells per retina, the mean number of cells at 1.0 and 3.5 mm from the optic disc was multiplied by the mean area of the retina. Five flat-mounted retinas with well-preserved borders were imaged under an EVOS Microscope (AMG); the area was measured using Image J software and averaged to obtain a mean value of retinal area.

### RGC axon labeling and counting

Cholera Toxin B subunit conjugated to Alexa488 (CTB-488, Invitrogen Inc.) was used as an anterograde tracer of RGC axons. Briefly, 4 µl of CTB-488 (0.2% diluted in PBS with 1% DMSO) was injected into the vitreous body two days before euthanasia. Animals were euthanized and perfused as described above, and the nerve segments were dissected to the level of the optic chiasm, and transferred to increasing sucrose solutions until 30%. Tissue was embedded in optimal cutting temperature (OCT, Tissue-Tek) and sectioned longitudinally on a cryostat (Leica Microsystems, Wetzlar, Germany) at 14 µm thickness. Optic nerve sections were rinsed with PBS, nuclei were counterstained with DAPI (4′,6-diamidino-2-phenylindole, 2.7 mg/ml, Sigma) and slides were mounted with Vectashield (Vector Laboratories). Nerve sections were observed under a Zeiss Axiovert 200 M microscope, and axon growth was analyzed by counting the number of axons labeled with CTB-488 extending from 0.25 to 2.0 mm from the crush site. Values were normalized by the formula described by Leon and colleagues [15] and expressed as the total number of axons per nerve at each distance from the lesion site.

### Histochemistry

Animals were perfused, and the eyes with attached nerves were dissected and transferred to sucrose solutions as in the previous item. Tissue was embedded in OCT and sectioned at 20 µm thickness. Tissue sections were rinsed with 0.1% PBST and incubated in 5% normal goat serum (Sigma-Aldrich Co) for 1 h at room temperature, followed by incubation with primary antibodies overnight at 4°C. Sections were then washed in 0.1% PBST and then incubated with secondary antibodies and TO-PRO-3 (1∶1000, Invitrogen Inc.) for 2 h at room temperature. Slides were rinsed in PBS and mounted with VectaShield (Vector Laboratories). Primary antibodies used were anti-dextran (mouse, 1∶500, StemCell Technologies), anti-IBA1 (rabbit, 1∶400, Wako Pure Chemical Industries, Osaka, Japan), anti-IL-1β (rabbit, 1∶100, Peprotech, London, UK) and anti-FGF-2 (mouse, 1∶200, Millipore). Secondary antibodies used were Alexa Fluor 488 goat anti-mouse IgG and Alexa Fluor 555 goat anti-rabbit IgG. Fluorescent samples were analyzed under a confocal microscope (Zeiss LSM 510 Meta). For the Prussian blue reaction and documentation, the procedure was similar to that described for cells on coverslips.

### Fluorescence intensity quantification

Fluorescence intensity quantification was performed two weeks after optic nerve crush. Briefly, retinal sections were immunostained with FGF-2 and IL-1β specific antibodies, and TO-PRO was used to stain nuclei, as described above. Three retinas from the untreated group and 3 retinas from the treated group were analyzed. For each retina, 9 Z stack images (1.3 µm thickness) from at least 3 different sections were randomly acquired with a confocal microscope (Zeiss LSM 510 Meta, ZEN 2009 software) under identical parameters for all the slides (microscope objective lens 40×1.3 NA, 16 bits images with a resolution of 512×512 pixels). Individual slices of each stack were analyzed using ZEN 2009 software. The area of the ganglion cell layer was outlined in each image, in the FGF-2 or IL-1β channel; the TO-PRO signal was used only for localization of the retinal layers. The average mean gray value per stack normalized by the vehicle injected group was used for statistical analysis.

### Statistical Analysis

For RGC survival analysis, the results for the crushed groups were expressed as a percentage of the mean of contralateral eyes and compared using an unpaired t-test. For analysis of axon regeneration, the number of axons in crushed groups was compared using an unpaired t-test at each distance from the crush site analyzed. For FGF-2 and IL-1β expression, the average mean gray value was normalized by vehicle injected group and an unpaired t-test was performed to compare vehicle and MSC injected groups. Results are displayed as the mean ± SEM. GraphPad Prism software was used for all statistical analyses.

## Results

### Characterization and distribution of MSC

**Figure 1:**
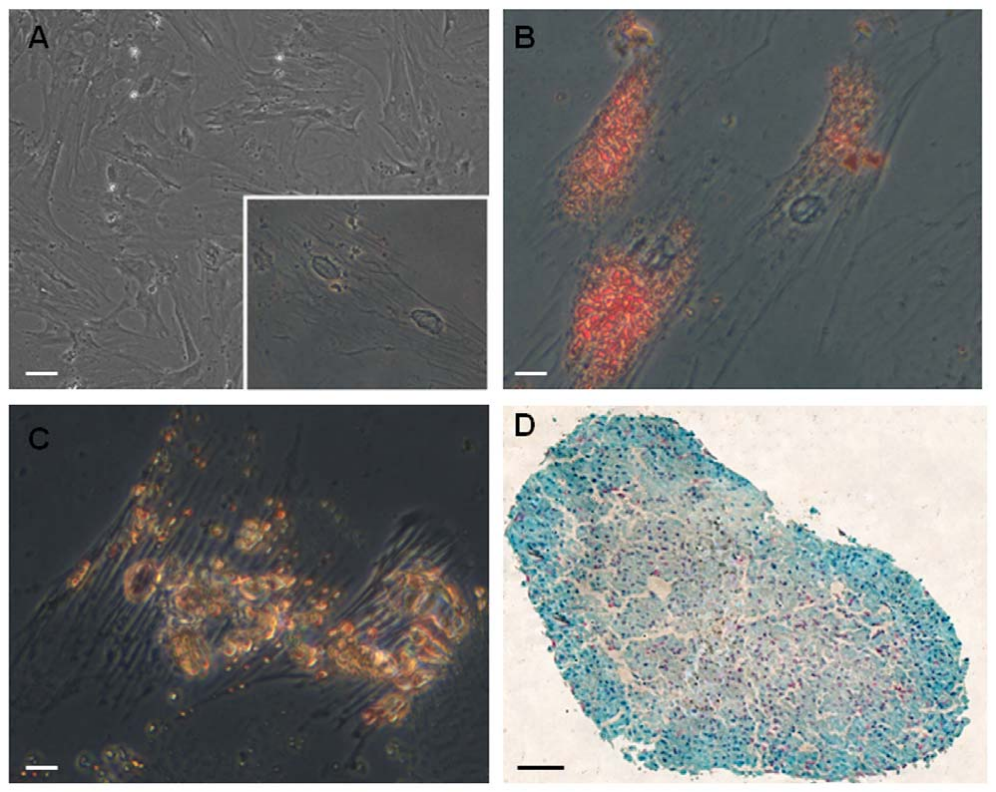
MSC differentiation in adipocytes, osteocytes and chondrocytes. (A) Cultured MSC have fibroblastoid morphology under phase contrast; inset shows adherent MSC in higher magnification. (B) Cells were cultivated in adipogenic medium, fixed and incubated with Oil Red O to identify lipid vacuoles (red staining). (C) Cells were cultured in osteogenic medium and calcium deposits were revealed with alizarin red (yellowish-brown staining). (D) Cells were cultivated in chondrogenic medium, forming a micromass; proteoglycans were stained with alcian blue and nuclei counterstained with Nuclear Fast Red. Scale bar: 50 µm (A), 12.5 µm (B,C), 50 µm (D).

**Figure 2:**
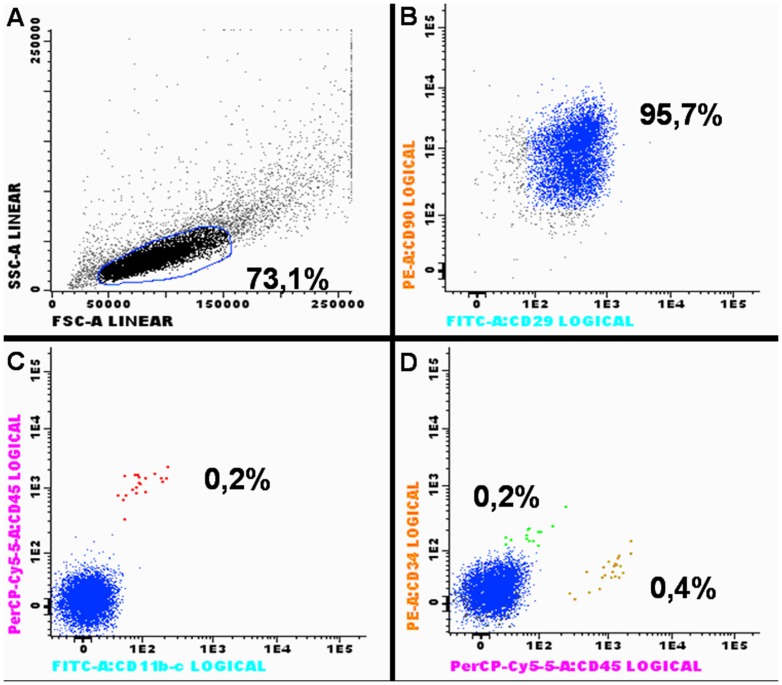
MSC immunophenotyping by flow cytometry. MSC were analyzed from passages 3 to 5, after being frozen and thawed. (A) Forward Scatter (FSC) and Side Scatter (SC) profiles of MSC. The gate in (A) was drawn to exclude doublets of cells. Ninety-five percent of the analyzed cells were positive for CD90 and CD29 (B), 99.8% were negative for CD45 and CD11b-c (C), 99.8% were negative for CD34, and 99.6% were negative for CD45 (D). These results are consistent with the phenotype of multipotent mesenchymal stem cells.

**Figure 3:**
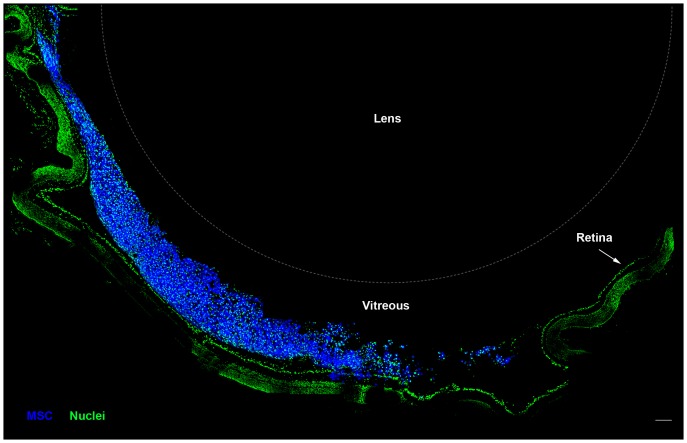
MSC are located in the vitreous body 1 day after optic nerve crush and cell transplantation. Photomontage of confocal images of a cryostat section through the eye. MSC (blue) were labeled with CellTrace prior to injection in the vitreous body, and were located mainly in this region 1 day after transplantation. Nuclei (green) in the retina and in the vitreous body were stained with Sytox Green. The lens is outlined with a dashed line. Scale bar: 100 µm.

The MSC phenotype was confirmed by the analysis of its differentiation potential in adipocytes, osteocytes and chondrocytes (Figure 1), as well as by the expression of CD90 and CD29 (Figure 2B), and the lack of expression of CD45, CD11b/c and CD34 (Figure 2C-D). For distribution analysis, MSC were labeled with CellTrace (Figure 3) and transplanted intravitreously after optic nerve crush. One day after the procedure, numerous labeled cells were found mainly in the vitreous body (Figure 3), in close proximity to the retina. Because fluorescent dyes can lose intensity with time, we used an additional method to track the transplanted cells *in vivo* and *ex vivo* for extended times, which is important information before the translation to a clinical therapy.

**Figure 4:**
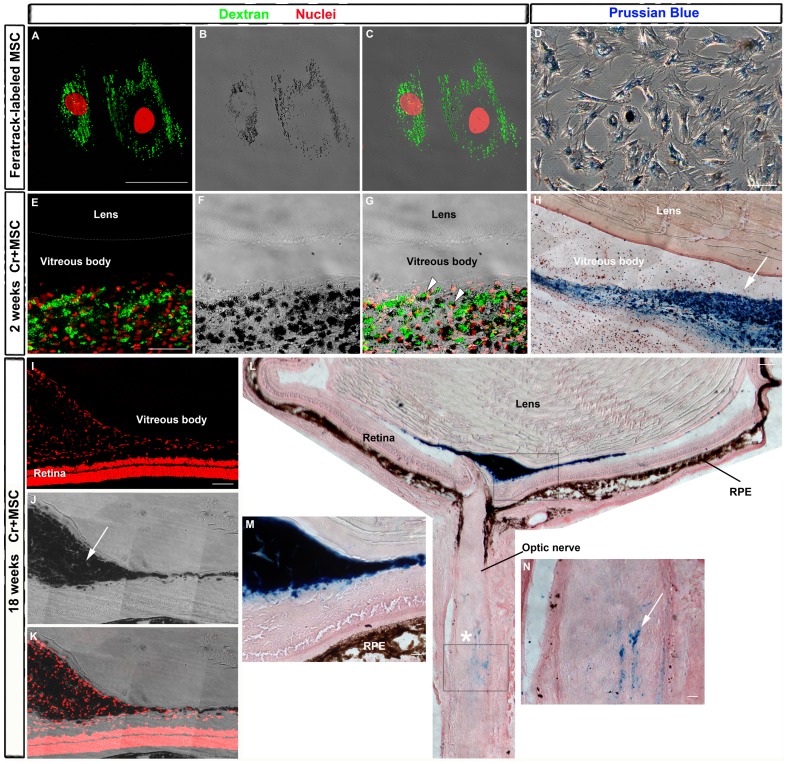
Detection of FeraTrack-labeled MSCs *in vitro* and after transplantation. A: Cells were labeled with FeraTrack for 4 h, fixed, and immunostained with anti-dextran antibody (green) to identify dextran-coated particles and nuclei stained with TOPRO (red) (A and C). Iron from SPION is visible as dark spots inside the cell (B-C) or after Prussian blue reaction (D). E-G: Sixteen days after nerve injury and MSC injection, these cells are found in the vitreous body as dextran-positive cells (green) with dark spots (arrowheads in G). Nuclei were stained with TOPRO (red, in E and F). H: Photomontage of transmitted light images of a cryostat section through the eye reacted with Prussian blue. Labeled cells are found mostly in the vitreous body (arrow). I-N: Eighteen weeks after nerve injury and cell transplantation, SPION are visible as dark spots by transmitted light (arrow in J) and also after Prussian blue reaction (L-N). I-K: Photomontage of confocal images from the eye; nuclei stained with TOPRO (red, in I and K). L: Photomontage of transmitted light images of the eye and proximal optic nerve after Prussian blue reaction. Iron was detected mainly in the vitreous body (blue in M), but a small amount was found also in the optic nerve (blue in N), close to the crush site (asterisk). (M,N) Higher magnification of upper (M) and lower (N) boxes in L. RPE, retinal pigmented epithelium. Scale bar: 50 µm.

MSC were labeled with SPION (FeraTrack), which can be visualized *in vivo* by MRI and also *ex vivo* by histochemical methods in the ocular tissues. In order to localize the MSC graft in the eye, we analyzed the presence of FeraTrack-labeled cells *ex vivo* at 2 and 18 weeks after nerve crush and cell transplantation. Figure 4A-D shows the presence of SPION in the MSC before transplantation. Since the particles are coated with dextran, it is possible to reveal them with a specific antibody (Figure 4A). In addition, the iron is easily seen as pigmented dots by transmitted light (Figure 4B) and is perfectly spatially correlated with dextran staining (Figure 4C). The iron in the SPION can also be revealed in FeraTrack-labeled MSC by the Prussian blue reaction (Figure 4D). Furthermore, we made cryostat sections of crushed nerves attached to MSC-injected eyes for dextran or Prussian blue staining. Pigmented cells positive for dextran were found in the vitreous body 2 weeks after cell transplantation (Figure 4E-G). A large amount of iron was also detected in the vitreous by the Prussian blue reaction (Figure 4H). After 18 weeks, iron-positive cells were still found in the vitreous (Figure 4I-K) and most of them were in the proximity of the optic disc, as revealed by Prussian blue staining (Figure 4L-M). To exclude the possibility of phagocytosis of iron-labeled MSC by inflammatory cells, we immunostained neighboring eye sections from the animals shown in Figure 4 with anti-IBA1 antibody, that identifies macrophages and microglia. IBA1 was expressed in the inner retinal layers, probably by resident microglia, but the vast majority of the cells found in the vitreous body were negative to IBA1 (Figure S2 in File S1). A small amount of iron was also found in the optic nerve in the vicinity of the crush site (Figure 4N).

**Figure 5:**
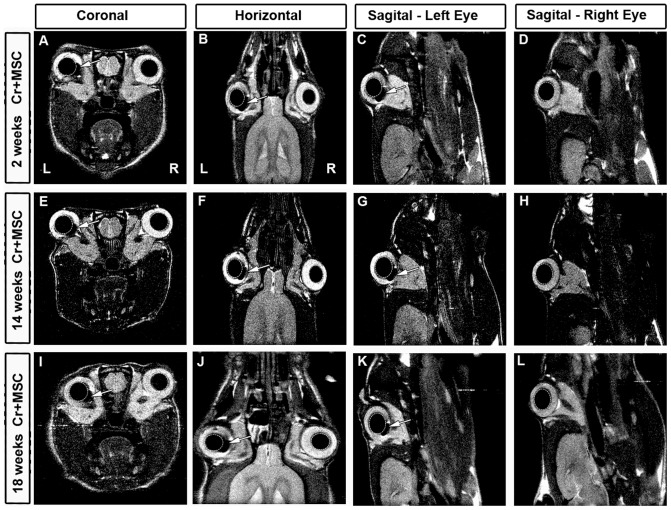
Detection of FeraTrack-labeled MSCs by *in vivo* MRI. MSC were labeled with Fera Track for 4 h and injected in the vitreous body of the left eye after optic nerve crush. (A-L) Representative images of *in vivo* MRI at coronal (A,E,I), horizontal (B,F,J), and sagittal planes (C,G,K; at left eye level and D,H,L; at right eye level) at different survival times. Arrows indicate hypointense (black) spots corresponding to FeraTrack-labeled cells in the vitreous body of the left eye. Labeled cells were found at 2 (A-C), 14 (E-G) and 18 weeks (I-K) after optic nerve crush and cell transplantation. The right eye was injected with the washing solution of the cells; no signal was detected (D,H,L and right hemisphere of coronal and horizontal images). The lenses are circled with dashed lines; right (R) and left (L) hemispheres.

Figure 5 A-D shows MRI images two weeks after unilateral optic nerve crush (left) and injection of FeraTrack-labeled MSC. The right eye was injected with the washing solution of the last cell centrifugation/washing. No signal of the washing solution injected in the right eye was detected. In contrast, the signal of the labeled cells was visible as a dark stain next to the lens in the left eye (arrow in Figure 5A-C). Furthermore, the signal of labeled cells remained in the left eye over time, up to 18 weeks after nerve crush and cell transplantation (Figures 5E-G and 5I-K). There was no evident reduction of the signal over the weeks analyzed, suggesting that the cells remained inside the eye for at least 18 weeks (compare Figures 5C, 5G and 5K).

Taken together, these results imply a long-term permanence of the MSC graft in the eye, mainly in the vitreous body. To assess the potential therapeutic effect of the transplanted cells, we analyzed RGC survival and axonal regeneration over time.

### MSC were neuroprotective after optic nerve crush

RGC survival was analyzed 16 and 28 days after optic nerve crush, quantifying the number of RGC immunostained with Tuj1 and Brn3a, at both 1.0 and 3.5 mm from the optic disc.

**Figure 6:**
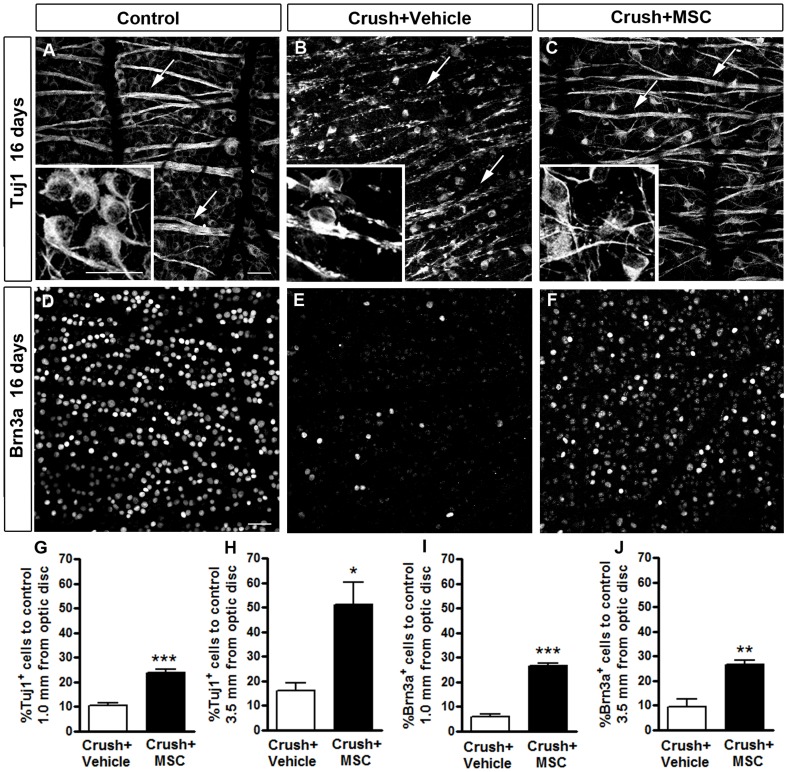
MSC transplantation increased RGC survival 16 days after optic nerve crush. A-F: Confocal images of flat-mounted retinas labeled with Tuj1 (A-C) or anti-Brn3a (D-F) antibodies. Insets in A-C are higher magnifications of the images to illustrate the morphology of the Tuj1-positive cells. In the control retina (contralateral eye) numerous cells are labeled with Tuj1 (A) and Brn3a (D), and axon bundles are intact (arrows in A). Sixteen days after optic nerve crush, there was a large reduction in the number of RGCs (B and E) and axon bundles were notably compromised, as revealed by Tuj1 staining (arrows in B). In the MSC-injected animals (C and F) the number of surviving RGCs was increased compared to the vehicle-injected ones, and Tuj1 staining in axon bundles was similar to the control (arrows in C). G-J: Quantification of RGC survival 16 days after nerve crush using Tuj1 (G, H) or Brn3a labeling (I, J). Results are displayed as mean ± SEM of the percentage of Tuj1+ or Brn3a+ cells relative to the control retina (contralateral eye). *P<0.05; **P<0.01; ***P<0.001. Scale bar in A-F: 50 µm.

Tuj1 recognizes neuronal βIII tubulin (Figure 6A) and is widely used as a RGC marker [10], [11], [17], [19], [27]. Indeed, one study demonstrated a correlation higher than 95% between Tuj1-positive cells in the ganglion cell layer and RGC retrogradely labeled with Fluorogold, after different experimental procedures [47].

Sixteen days after optic nerve crush, we observed a reduction in the number of cell bodies immunostained with Tuj1 (inset in Figure 6B) and also degeneration of the nerve fibers inside the retina (arrows in Figure 6B), compared to the control retina (Figure 6A). The reduction in the number of RGC after optic nerve crush was less evident after MSC transplantation (6C). In addition, the optic fibers in the retina were more preserved (arrows in Figure 6C) and qualitatively similar to those observed in the control retinas (Fig 6A). Quantitative analyses showed that the number of Tuj1-positive cells in the vehicle-injected group was reduced to 10.34% and 16.16% of the number in the control group, at 1.0 and 3.5 mm from the optic disc, respectively. Treatment with MSC increased these percentages to 23.61% (Figure 6G) and 51.09% (Figure 6H), respectively, which represent 1.3- and 2.2-fold increases of the MSC-treated group over the group treated with the vehicle alone.

We also studied the survival of RGC using immunostaining for Brn3a, a transcription factor that is a member of the class Brn3 of POU-homeodomain factors [48]. Brn3a has been reported as a reliable marker of adult rat RGC in both naïve and injured retinas [49]–[51]. Sixteen days after lesion, there was a very large decrease in the number of Brn3a-positive cells in the retina (Figure 6E), compared to control retinas (Figure 6D). In the MSC-treated group, the decrease in RGC number was less dramatic than in the untreated group (Figure 6F). The quantitative analyses revealed that in the vehicle-injected group, the number of Brn3a-positive cells was reduced to 5.78% and 9.31% of the control, at 1.0 and 3.5 mm from the optic disc, respectively. MSC injection increased these percentages to 26.5% (Figure 6I) and 26.4% (Figure 6J), respectively, which represents 3.5- and 1.8-fold increases of the MSC-treated group over the group treated with the vehicle alone.

**Figure 7:**
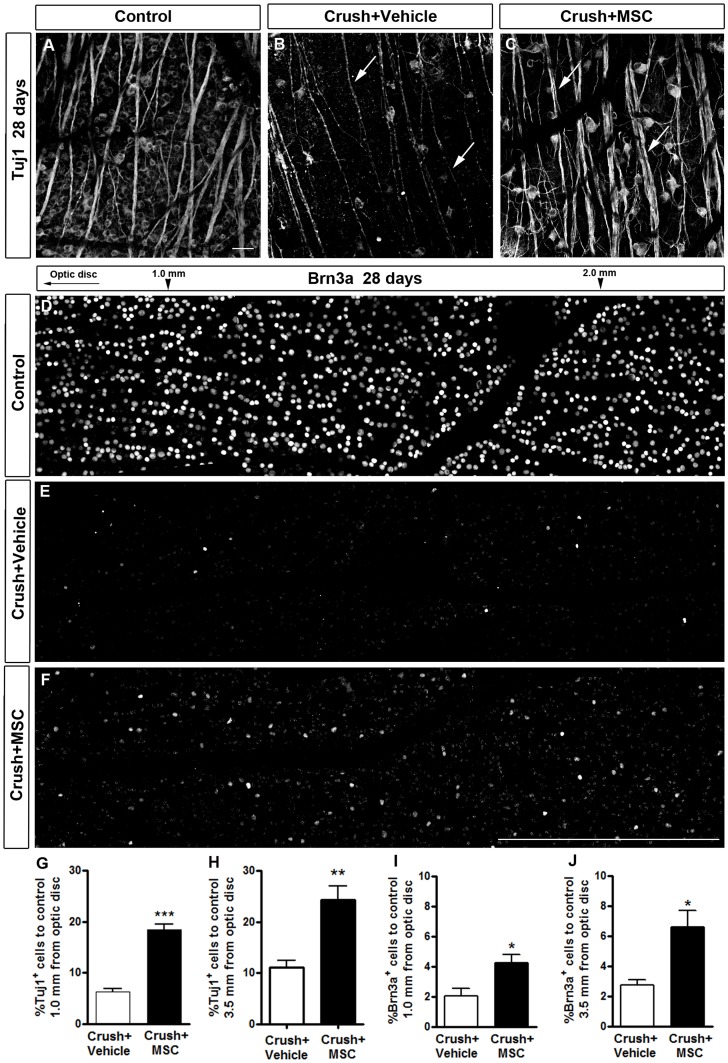
MSC transplantation increased RGC survival 28 days after optic nerve crush. A-C: Confocal images of flat-mounted retinas labeled with Tuj1 antibody (A-C). D-F: Photomontage of confocal images of flat-mounted retinas labeled with Brn3a antibody. A, D: Control retina (contralateral eye). B, E: Twenty-eight days after optic nerve injury, there was a large reduction in the number of RGCs, and axon bundles were thinner (arrows in B) in the vehicle-treated group compared to control. C, F: In MSC-injected animals, the number of surviving RGCs increased compared to vehicle-injected animals, and axon bundles were similar to control (arrows in C). G-J: Quantification of RGC survival 28 days after nerve crush at 1.0 mm or 3.5 mm from the optic disc, using Tuj1 (G, H) or Brn3a labeling (I, J). Results are displayed as mean ± SEM of the percentage of Tuj1+ or Brn3a+ cells relative to the control retina (contralateral ye). *P<0.05; **P<0.01; ***P<0.001. Scale bar: 50 µm (A-C); 500 µm (D-F).

Twenty-eight days after optic nerve crush, we observed a very large reduction in the number of Tuj1-positive cells and thinning of nerve fibers in the retina in the vehicle-injected group (arrows in Figure 7B), compared to the control (Figure 7A). In the MSC-injected group the number of Tuj1-positive cells (Figure 7C) was also reduced compared with the control but was larger than in the vehicle-injected group. Axon bundles are also more preserved in the MSC treated than in the vehicle injected group (arrows in Figure 7B-C). The quantitative analyses revealed that the percentage of Tuj1-positive cells in the vehicle-injected group was 6.28% and 11.0% of control, at 1.0 and 3.5 mm from the optic disc, respectively. MSC injection increased these percentages to 18.4% (Figure 7G) and 24.3% (Figure 7H), respectively, which represent 1.9- and 1.2-fold increases of the MSC-treated group over the group treated with the vehicle alone.

The number of Brn3a-positive cells was dramatically reduced 28 days after nerve crush in the vehicle-injected group (Figure 7E), compared to the control (Figure 7D). This reduction was attenuated when the animals were treated with MSC (Figure 7F). The quantitative analysis showed that the percentage of Brn3a-positive cells in the vehicle-injected group was only 2.03% and 2.73% of the number in the control, at 1.0 and 3.5 mm from the optic disc, respectively. MSC injection increased these percentages to 4.24% (Figure 7I) and 6.61% (Figure 7J), respectively, which represent 1.1- and 1.4-fold increases of the MSC-treated group over the group treated with the vehicle alone. The quantification of Tuj1- and Brn3a-positive cells and the estimated number of RGC per retina are summarized in Table S1 in File S1. These results clearly indicate that intravitreous MSC transplantation significantly promoted long-term neuroprotection of RGC after a severe optic nerve crush.

### MSC promoted axonal regeneration after optic nerve crush

**Figure 8:**
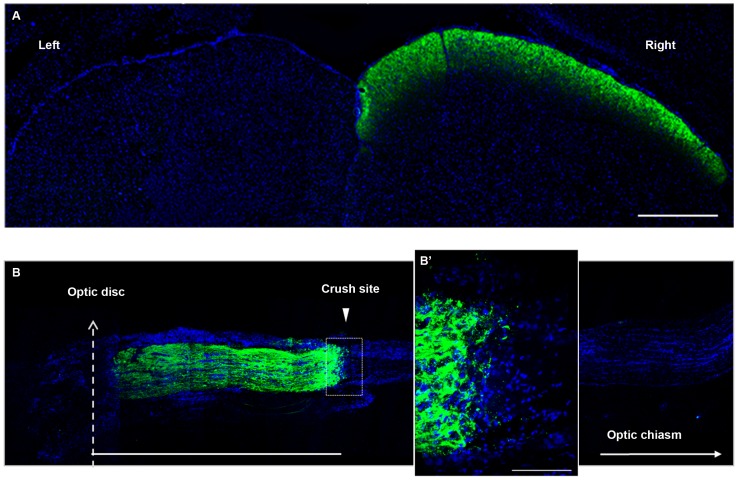
Anterograde labeling with CTB-488. (A) Photomontage of images of the superior colliculus of a rat 2 days after intravitreous injection of CTB-488 (green). In the absence of optic nerve injury, the tracer is transported from the left eye through the optic nerve until it reaches the right superior colliculus. (B) Photomontage of images of a nerve 4 days after injury. CTB-488 positive axons (green) do not extend farther into the crush site; B′ shows higher magnification. Nuclei (blue) were stained with DAPI. Scale bars: 250 (A), 1000 (B) and 100 (B′) µm.

**Figure 9:**
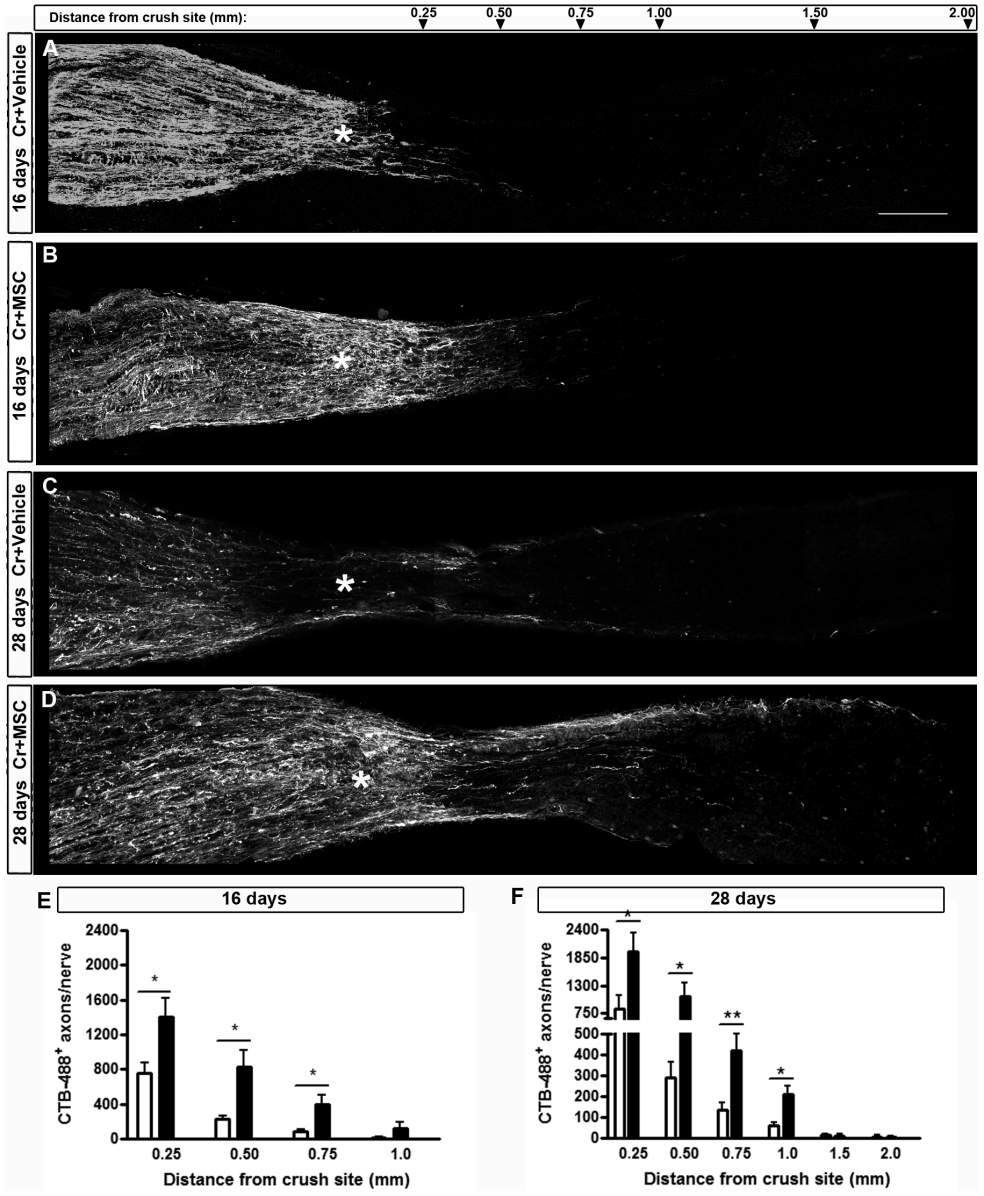
MSC transplantation increased RGC axon regeneration 16 and 28 days after optic nerve crush. A-D: Photomontage of confocal images of optic nerve sections to illustrate axonal outgrowth. CTB-488 was injected into the vitreous body 2 days prior to euthanasia to label the axons. Sixteen days after nerve crush (A,B,E) only a few axons had crossed the lesion site (asterisk) in the vehicle-injected animals (A) and this number was larger in the MSC-injected animals (B). Twenty-eight days after optic nerve injury (C,D,F), the MSC-injected group had an even larger number of axons regenerating beyond the crush site, compared to the vehicle-injected group. E, F: Quantification of CTB-488^+^ axons per nerve at different distances from the crush site (from 0.25 to 2.0 mm). Results are displayed as mean ± SEM. *P<0.05; **P<0.01. Scale bar: 200 µm.

To assess the number of axons that are able to regenerate after the crush site, we labeled them with CTB-488 injected into the vitreous body. The toxin is anterogradely transported from the retinal ganglion cells to the superior colliculus, through undamaged nerves (Figure 8A). In the crushed nerves, we counted the positive fibers at 0.25, 0.50, 0.75, 1.00, 1.50 and 2.00 mm distal from the crush site (Figure 9). In our model, optic nerve crush damages all axons; four days after the lesion, virtually all axons had CTB-488 transport interrupted at the injury site (Figure 8B). Sixteen days after injury, a few axons were able to regenerate farther from the crush site in the vehicle-injected animals (Figure 9A), and MSC treatment significantly increased (by 3.2 fold) the number of axons extending up to 0.75 mm from the injury site (Figure 9B, E; P<0.001; 94.82±21.54 axons/nerve in the vehicle-injected group and 401.9±109.1 axons/nerve in the MSC-injected group). After 28 days, the axons had grown further in both groups (Figure 9C-D), but the MSC-injected group showed a 2.0-fold increase in the number of axons extending up to 1.00 mm from the crush site (Figure 9F; P<0.05; 59.37±17.00 axons/nerve in the vehicle-injected group and 176.8±38.95 axons/nerve in the MSC-injected group). The mean of axons per nerve at each distance is given in Table S2 in File S1. These results suggest that MSC therapy increased the number of axons that cross the injury site and stimulated them to regenerate over longer distances compared with vehicle-treated eyes. In addition, this regenerative effect of the cell therapy was sustained overtime, as seen at 16 and 28 days after injury.

### MSC injection upregulated FGF-2 and IL-1β in the ganglion cell layer

**Figure 10:**
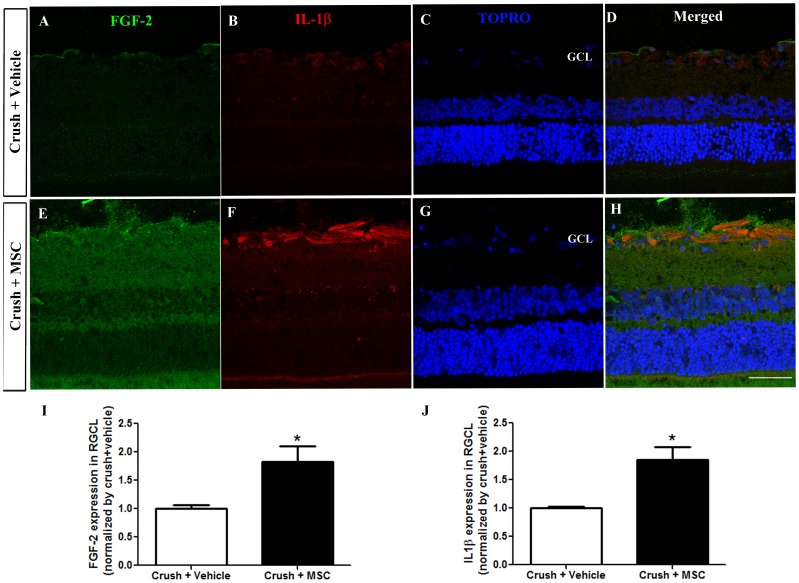
MSC transplantation increases FGF-2 and IL-1β expression in the retinal ganglion cell layer. (A-H) Confocal images of FGF-2 and IL-1β expression in retinas from vehicle injected (A-D) and MSC treated (E-H) groups. TOPRO (C, G) was used for nuclei staining. Images are representative of 3 animals per experimental condition. Scale Bar: 20 µm. (I, J) Graphs show the average mean gray value of Z stack images normalized by the vehicle injected group. *p<0.05, unpaired t-test. RGCL: retinal ganglion cell layer. Scale bar: 50 µm.

We analyzed the expression of FGF-2 (Figure 10A-D) and IL-1β (Figure 10E-H) in the retinal ganglion cell layer of the saline injected (n = 3) and MSC treated (n = 4) groups, using specific antibodies. FGF-2 expression increased significantly (P<0.05, Figure 10I) in the MSC treated group (1.827±0.2767, ratio to vehicle injected group). IL-1β was also significantly more expressed (P<0.05, Figure 10J) in the MSC treated group (1.854±0,2278, ratio to vehicle injected group).

## Discussion

Optic nerve crush induced drastic RGC degeneration, which has been widely associated to programmed cell death [52], [53]. Several apoptotic effectors such as caspase-3 and -9, Bax, Bcl-2 and p38 MAP kinase are modulated in the retina after optic nerve injury [54]–[58] and active caspase-3 is expressed by terminal transferase-mediated dUTP nick-end labelling (TUNEL)-positive rat RGC after axotomy [55]. We observed a more than 90% reduction in the number of Tuj1 and Brn3a positive cells in the retina after optic nerve crush, corroborating the results of several other studies on RGC degeneration after axonal damage [51], [59], [60].

In previous studies we have shown that treatment with bone marrow mononuclear cells (BMMC) reduces RGC death and increases nerve regeneration 2 weeks after optic nerve crush [23]. BMMC are a mixed population containing different types of myeloid and lymphoid cells and also stem/progenitor cells such as MSC [31], [61]. One could argue that cell therapies with mixed populations of cells have the advantage of affecting several potential targets at the same time. On the other hand, identifying the possible therapeutic effects of each transplanted cell type is an intricate task. Here we investigated the long-term effects of MSC therapy, a purified population of bone marrow-derived cells, in the same model of optic nerve injury that we have studied previously. All cells used in this study were once frozen and thawed, which facilitates their translation into a putative clinical therapy. Another advantage of the MSC is that it is possible to increase their numbers in order to obtain uniform populations of well-characterized cells, and therefore, any therapy-derived effect can be narrowed to a single cell type.

Furthermore, during the last 10 years, preclinical studies have shown that MSC transplantation is a promising approach to treat several neurological diseases [62], such as amyotrophic lateral sclerosis [63], [64], Alzheimer's disease [65], [66], Parkinson's [67], spinal cord injury [68], stroke [69], [70] and multiple sclerosis [71], [72]. In experimental models of these diseases, the administration of MSC through different routes was able to increase the survival of motor neurons [73], [74], reduce the accumulation of amyloid-β (Aβ) in the brain [65], reduce the loss of dopaminergic neurons [75], improve functional outcome [76], [77] and reduce demyelination [78], [79]. Some studies have associated the beneficial effects with the increase of neurotrophins in the injured tissue [80], [81], and, importantly, many of them have suggested that MSC can control the inflammatory activity associated with tissue degeneration [62], e.g., by promoting an alternative microglial activation [82], [83] or controlling the infiltration of inflammatory cells into the central nervous system [71].

In general, intraocular transplanted cells do not migrate inside the neural retina [42]–[44] and we found that several weeks after Feratrack-labeled MSC transplantation, the iron from Feratrack was localized in the vitreous body, which is free of resident cells. The MRI signal intensity was not decreased overtime and several nuclei were identified in the regions with iron up to 18 weeks after crush and cell transplantation (the longest period of time that we have analyzed). Although we cannot exclude the possibility that monocytes/macrophages invade the vitreous, these cells have a very short life span and for that reason it is very unlikely that the iron signal and nuclei could be associated to inflammatory cells and not to the transplanted MSC. Indeed, the vast majority of the cells found in the vitreous did not express IBA1, supporting the permanence of the MSC graft. This suggests that MSC may exert a more continuous and long-term effect than canonical transitory approaches, e.g., a single injection of a drug or trophic factor. Furthermore, we also expected to have a sustained effect compared to BMMC transplantation, since these cells are no longer found in the eye two weeks after they were injected [39].

The presence of MSC in the eye for several days could be directly responsible for the therapeutic effect observed in our study. We have found that in the treated group, the number of RGC revealed with either Tuj1 or Brn3a was at least twice as large as in the vehicle group, at both 16 and 28 days after injury. Notably, the percentage of Brn3a-positive cells was smaller than that of Tuj1-positive cells in the crushed groups. This difference can be explained by the rapid loss of Brn3a in injured RGC [51] and the presence of displaced amacrine cells [84] labeled with Tuj1 in the ganglion cell layer. In addition, in both experimental groups there was a decrease in RGC survival from 16 to 28 days after injury (Figure S1 in File S1), which was expected, since the degeneration is progressive; but suggests that not all of the cells that were protected by cell therapy at 16 days were effectively rescued. However, both Tuj1 and Brn3a markers had significantly different percentages between the MSC- and vehicle-injected groups, indicating a higher RGC survival due to cell therapy. Together with the increase in the number of regenerating axons beyond the crush site, our data also suggests that in the MSC-treated group there is a large number of RGC and/or that the protected RGC were capable of extending longer axons than in the vehicle-injected group. A longer follow-up period after injury would reveal whether these beneficial MSC effects are sustained.

The mechanisms of action of MSC after transplantation are still elusive but it is important to consider that intravitreally delivered MSC are confined to the site of injection and their effect is rather local than systemic, which would be the case if the cells were injected intravenously. In the vitreous body, MSC are in close proximity to the damaged retina, favoring paracrine activity and the modulation of host cells. Indeed, we have seen that MSC *in vitro* express trophic factors such as fibroblast growth factor 2 (FGF-2), vascular endothelial growth factor (VEGF) and ciliary neurotrophic factor (CNTF) (data not shown), and it was shown that MSC produce VEGF, brain-derived neurotrophic factor (BDNF), nerve growth factor (NGF) and hepatocyte growth factor (HGF) when cultured with injured brain extracts [85], [86]. In a glaucoma model and after ischemia/reperfusion of the retina, MSC increase RGC survival and the expression of CNTF and FGF-2 in the retina [42], [43]. FGF-2 is one of the most studied growth factors from the point of view of its neuroprotective effects[87] and our results showed that MSC upregulated FGF-2 in the retinal ganglion cell layer, similar to what we observed after therapy with bone marrow mononuclear cells[23].

In addition to the modulation of trophic factors, MSC have immunomodulatory properties that were identified with reports that MSC could arrest T cell proliferation [88], [89] and this characteristic has been increasingly explored as a potential approach to treat neuroinflammatory disorders [90]. Indeed, intravenous or intracerebral injection of MSC ameliorated the clinical course of experimental autoimmune encephalomyelitis (EAE) in mice by inducing T cell anergy and reducing lymphocytic infiltration [71], [91]. MSC have been suggested to inhibit not only T lymphocytes but also other immune cells such as B cells, dendritic cells and natural killer cells [92]. There are also evidences that MSC induce an alternative activation of microglia into an anti-inflammatory phenotype, decreasing neuronal death after ischemia and favoring the clearance of Aβ in a transgenic model of Alzheimer's disease [36], [66]. Importantly, when cultivated with T cell blasts or T cell hybridomas that do not produce inflammatory cytokines in the absence of antigen stimulation, MSC were not immunosuppressive [93]. Indeed, there are cumulative evidences that MSC must be activated or "licensed" by inflammatory pro-cytokines, in particular IL-1β, interferon (IFN)-γ and TNF-α, in order to exert their immunosuppressive effects [92]–[94]. In this study, we observed that animals treated with MSC had an increased expression of IL-1β in the retina, specifically in the RGC layer, suggesting that MSC created a pro-inflammatory environment. It is possible that phagocytosis of eventually dead MSC had resulted in the release of chemokines and cytokines, including IL-1. Because MSC are primed by inflammatory cytokines, it is likely that the retinal microenvironment produced by optic nerve crush and MSC themselves would be responsible for "licensing" the remaining alive MSC in order to secrete trophic factors that could protect the neurons and/or modulate the local microglia and other retinal glial cells. Recently it was shown that IL-1 and other pro-inflammatory cytokines may support neuronal survival [95]–[97] and that inflammatory stimulation induced either by lens injury [13], [15], [98], intravitreal injections of Zymozan [17], [99], crystallins [100] or toll-like receptors 2 agonists [101] transforms RGC into a regenerative state, enabling these neurons to survive and grow axons over the inhibitory environment of the injured optic nerve. The inflammatory beneficial effects were associated with the release of oncomodulin by recruited macrophages [16], [18] and neutrophils [102], as well as with the modulation of astrocyte-derived CNTF [103], leukemia inhibitory factor (LIF) [19], interleukin-6 (Il-6) [104] and consequent activation of several signaling pathways in the retinal cells. These include Janus kinase/signal transducers and activation of transcription-3 (JAK/STAT3) and phosphatidylinositide 3-kinase/protein kinase B/mammalian target of rapamycin (PI3K/AKT/mTOR) signaling cascades [105], [106]. Recent studies targeted these pathways and obtained impressive results on RGC survival and axon regeneration through the optic nerve [10]–[12]. IL-1β induces phosphorilation of Akt and activation of PI3K/AKT [107]–[109] and intravitreal injections of this cytokine rescued axotomized RGC from retrograde cell death [110]. These studies introduce a possible neuroprotective role of IL-1β and link ocular inflammation to neuronal survival and regeneration. We speculate that MSC ability to interact with immune cells might direct the complex milieu created by optic nerve damage into a protective environment for RGC, and our results suggest that FGF-2 and IL- 1β participate in the trophic and immunomodulatory effect that could promote the activation of signaling cascades such as PI3K/AKT that favor cell survival and regeneration.

## Conclusions

We have demonstrated that MSC promote long-term neuroprotection and axon regeneration after optic nerve crush. The prolonged effect, compared to our previous results with BMMC therapy, may be due to a longer permanence of the graft. MSC mechanisms of action may include the upregulation of trophic factors, such as FGF-2, and modulation of neuroinflammation by increasing the expression of cytokines such as IL-1β in the damaged tissue. Further efforts in the understanding of MSC mechanisms of action in the visual system must be made to avoid their application as an empirical therapy, without full awareness of their proper effects in each biological environment [111]. Our results support the therapeutic potential of MSC in the central nervous system, but further studies are necessary to identify additional factors that may be released and the modulated cells, once MSC are injected and persist for a long period of time in damaged neuronal tissue.

## Supporting Information

File S1 Figure S1, RGC survival over time. Graphs show the percentage of Tuj1 (A,B) or Brn3a (C,D) positive cells compared to the control (contralateral eyes), at 1.0 mm (A,C) and 3.5 mm (B,D) from the optic disc. Although there is a clear and significant neuroprotective effect of the MSC (asterisks), the percentage of Tuj1- and Brn3a-positive cells decreased at both distances from the optic disc from 16 to 28 days after optic nerve crush. Figure S2, The majority of the cells found in the vitreous body do not express IBA1. (A) Confocal image of an eye section immunostained with a specific antibody to IBA1 (red), 18 weeks after MSC transplantation. (B) Differential interference contrast microscopy image; iron reflection is seen as a dark area in the image. (C) Merged images. IBA1-positive cells were found in the inner retinal layers (arrows). In the vitreous body, the vast majority of nuclei (blue) was not associated with IBA1 expression. Rare IBA1-positive cells were present in the vitreous body (arrowhead). Nuclei were stained with TOPRO3. RPE: retinal pigmented epithelium. Table S1, Number of Tuj1- and Brn3a-positive cells in the retina. Table shows the number of cells per square millimeter of retina, SEM, and the estimated number of cells per retina at 16 and 28 days after injury. Sixteen days after injury, the number of Tuj1-positive cells is 2.7-fold increased in the treated group, whereas the number of Brn3a-positive cells increased 3.8-fold. Twenty-eight days after injury, the number of Tuj1-positive cells increased 2.5-fold in the treated group, whereas the number of Brn3a- positive cells increased 2.2-fold. The number of experiments (n) is indicated at each point. Table S2, Number of axons extending from 0.25 to 2.0 mm from the crush site. Table shows the mean and SEM of axons per nerve at each distance from the crush site at 16 and 28 days after injury. Sixteen days after injury, the number of axons at 1.0 mm from the crush site increased 4.7-fold in the treated group; whereas at 28 days after injury, the number of axons increased 3.0-fold in the treated group. The number of experiments (n) is indicated at each point.
(PDF)
