# Supplementary material for: Distribution of Mesenchymal Stem Cells and Effects on Neuronal Survival and Axon Regeneration after Optic Nerve Crush and Cell Therapy
Source: PLoS One. 2014 Oct 27;9(10):e110722. doi: 10.1371/journal.pone.0110722 (PMC4210195; doi:10.1371/journal.pone.0110722)
Supplement: File S1 — Figure S1, RGC survival over time. Graphs show the percentage of Tuj1 (A,B) or Brn3a (C,D) positive cells compared to the control (contralateral eyes), at 1.0 mm (A,C) and 3.5 mm (B,D) from the optic disc. Although there is a clear and significant neuroprotective effect of the MSC (asterisks), the percentage of Tuj1- and Brn3a-positive cells decreased at both distances from the optic disc from 16 to 28 days after optic nerve crush. Figure S2, The majority of the cells found in the vitreous body do not express IBA1. (A) Confocal image of an eye section immunostained with a specific antibody to IBA1 (red), 18 weeks after MSC transplantation. (B) Differential interference contrast microscopy image; iron reflection is seen as a dark area in the image. (C) Merged images. IBA1-positive cells were found in the inner retinal layers (arrows). In the vitreous body, the vast majority of nuclei (blue) was not associated with IBA1 expression. Rare IBA1-positive cells were present in the vitreous body (arrowhead). Nuclei were stained with TOPRO3. RPE: retinal pigmented epithelium. Table S1, Number of Tuj1- and Brn3a-positive cells in the retina. Table shows the number of cells per square millimeter of retina, SEM, and the estimated number of cells per retina at 16 and 28 days after injury. Sixteen days after injury, the number of Tuj1-positive cells is 2.7-fold increased in the treated group, whereas the number of Brn3a-positive cells increased 3.8-fold. Twenty-eight days after injury, the number of Tuj1-positive cells increased 2.5-fold in the treated group, whereas the number of Brn3a- positive cells increased 2.2-fold. The number of experiments (n) is indicated at each point. Table S2, Number of axons extending from 0.25 to 2.0 mm from the crush site. Table shows the mean and SEM of axons per nerve at each distance from the crush site at 16 and 28 days after injury. Sixteen days after injury, the number of axons at 1.0 mm from the crush site increased 4.7-fold in the treated g [file pone.0110722.s001.pdf]

Support Information

	16 days								
	**Control (fellow eye)**			**Cr+Vehicle**			**Cr+MSC**		
**1.0mm**	**cells/mm^2**	**SEM**	**n**	**cells/mm^2**	**SEM**	**n**	**cells/mm^2**	**SEM**	**n**
Tuj1$^+$	1516	59.95	10	156.8	19.30	8	358.0	21.39	8
Brn3a$^+$	2602	112.2	6	150.3	33.35	3	689.3	26.17	4
**3.5mm**	**cells/mm^2**	**SEM**	**n**	**cells/mm^2**	**SEM**	**n**	**cells/mm^2**	**SEM**	**n**
Tuj1$^+$	996.9	69.43	9	161.1	31.81	4	509.4	90.55	4
Brn3a$^+$	1288	106.8	9	119.9	39.97	4	340.6	24.58	4
	**Cells/retina** *(mean of 1.0 and 3.5 mm x mean retinal area)*								
	**Control (fellow eye)**			**Cr+Vehicle**			**Cr+MSC**		
Tuj1$^+$	79156			10014			27323		
Brn3a$^+$	122535			8511			32442		
	**28 days**								
	**Control (fellow eye)**			**Cr+Vehicle**			**Cr+MSC**		
**1.0mm**	**cells/mm^2**	**SEM**	**n**	**cells/mm^2**	**SEM**	**n**	**cells/mm^2**	**SEM**	**n**
Tuj1$^+$	1416	47.01	20	88.88	8.337	9	260.4	16.60	8
Brn3a$^+$	2475	69.13	13	50.27	12.11	6	105.0	13.41	6
**3.5mm**	**cells/mm^2**	**SEM**	**n**	**cells/mm^2**	**SEM**	**n**	**cells/mm^2**	**SEM**	**n**
Tuj1$^+$	1072	57.28	8	118	15.41	7	260.9	28.94	8
Brn3a$^+$	1187	51.83	8	32.36	4.049	5	78.43	12.74	6
	**Cells/retina** *(mean of 1.0 and 3.5 mm x mean retinal area)*								
	**Control (fellow eye)**			**Cr+Vehicle**			**Cr+MSC**		
Tuj1$^+$	78372			6517			16421		
Brn3a$^+$	115353			2603			5778		

Table S1. Number of Tuj1- and Brn3a-positive cells in the retina. Table shows the number of cells per square millimeter of retina, SEM, and the estimated number of cells per retina at 16 and 28 days after injury. Sixteen days after injury, the number of Tuj1-positive cells is 2.7-fold increased in the treated group, whereas the number of Brn3a-positive cells increased 3.8-fold. Twenty-eight days after injury, the number of Tuj1-positive cells increased 2.5-fold in the treated group, whereas the number of Brn3a-positive cells increased 2.2-fold. The number of experiments (n) is indicated at each point.

Distance from crush site	16 days					
	Cr+Vehicle			Cr+MSC		
	**Mean of axons per nerve**	**SEM**	**n**	**Mean of axons per nerve**	**SEM**	**n**
0.25 mm	766.5	120.8	8	1408	214.8	8
0.50 mm	231.2	40.72	8	827.1	196.9	7
0.75 mm	94.82	21.54	8	401.9	109.1	8
1.00 mm	27.03	11.59	8	126.8	69.14	7
	**28 days**					
	**Cr+Vehicle**			**Cr+MSC**		
	**Mean of axons per nerve**	**SEM**	**n**	**Mean of axons per nerve**	**SEM**	**n**
0.25 mm	839.1	271.3	7	1970	373.0	7
0.50 mm	291.4	76.83	7	1080	266.0	7
0.75 mm	134.4	37.95	7	421.7	78.87	7
1.00 mm	59.37	17.00	7	176.8	38.95	7
1.50 mm	14.98	6.147	7	13.07	6.659	7
2.00 mm	8.909	8.909	7	6.929	6.266	7

Table S2. Number of axons extending from 0.25 to 2.0 mm from the crush site. Table shows the mean and SEM of axons per nerve at each distance from the crush site at 16 and 28 days after injury. Sixteen days after injury, the number of axons at 1.0 mm from the crush site increased 4.7-fold in the treated group; whereas at 28 days after injury, the number of axons increased 3.0-fold in the treated group. The number of experiments (n) is indicated at each point.

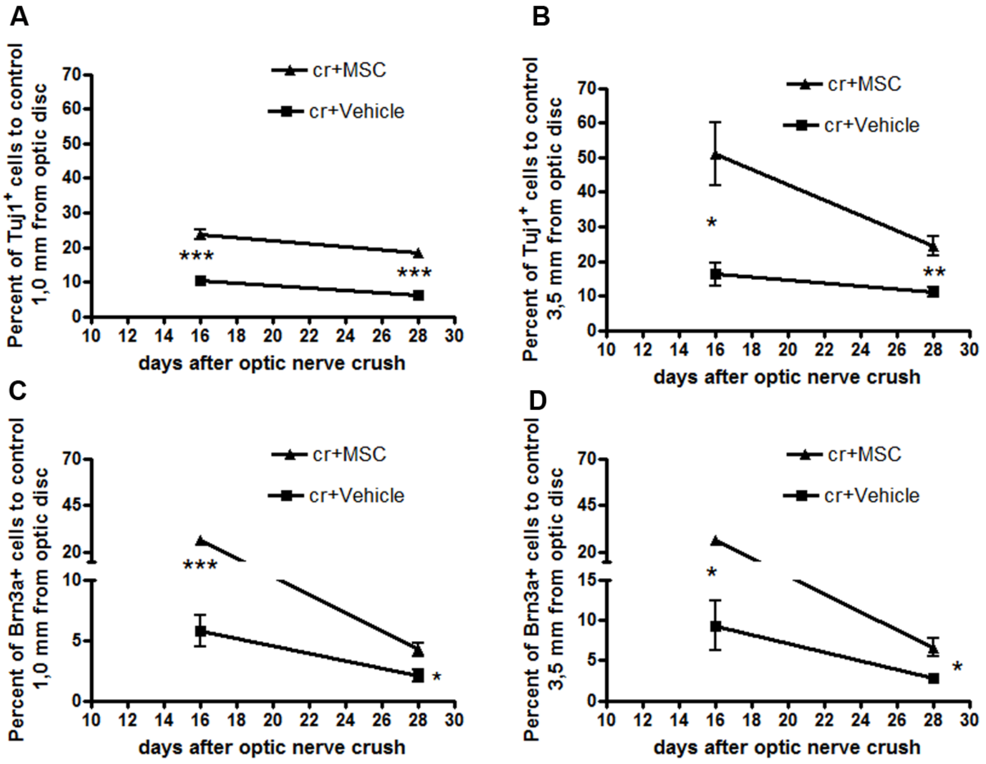

Figure S1. RGC survival over time. Graphs show the percentage of Tuj1 (A,B) or Brn3a (C,D) positive cells compared to the control (contralateral eyes), at 1.0 mm (A,C) and 3.5 mm (B,D) from the optic disc. Although there is a clear and significant neuroprotective effect of the MSC (asterisks), the percentage of Tuj1- and Brn3a-positive cells decreased at both distances from the optic disc from 16 to 28 days after optic nerve crush.

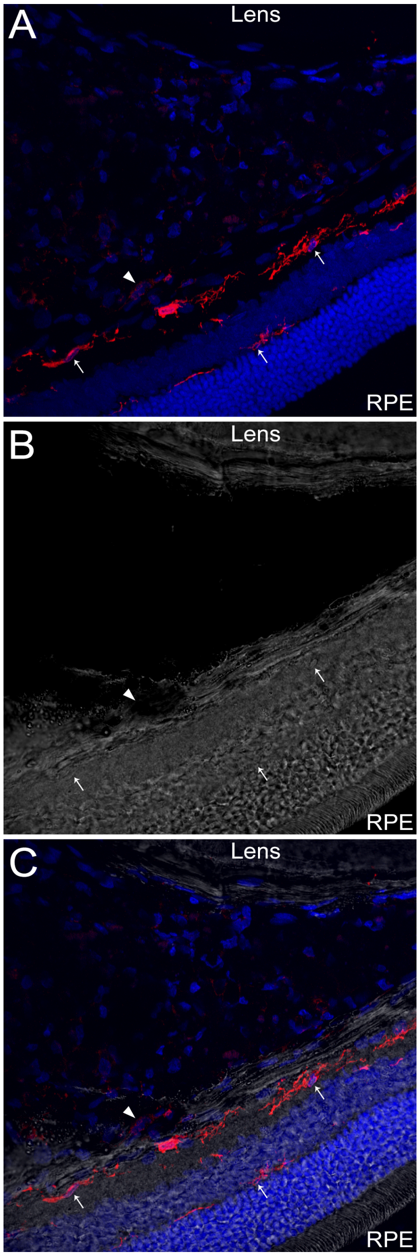

Figure S2. The majority of the cells found in the vitreous body do not express IBA1. (A) Confocal image of an eye section immunostained with a specific antibody to IBA1 (red), 18 weeks after MSC transplantation. (B) Differential interference contrast microscopy image; iron reflection is seen as a dark area in the image. (C) Merged images. IBA1-positive cells were found in the inner retinal layers (arrows). In the vitreous body, the vast majority of nuclei (blue) was not associated with IBA1 expression. Rare IBA1-positive cells were present in the vitreous body (arrowhead). Nuclei were stained with TOPRO3. RPE: retinal pigmented epithelium.
